# The Right to Oncological Oblivion: A Legislative Response to Cancer Survivor Discrimination in Italy

**DOI:** 10.3390/healthcare12161665

**Published:** 2024-08-21

**Authors:** Gianpiero D’Antonio, Ginevra Bolino, Letizia Sorace, Gianpietro Volonnino, Lavinia Pellegrini, Nicola Di Fazio, Paola Frati

**Affiliations:** Department of Anatomical, Histological, Forensic and Orthopedic Sciences, Sapienza University of Rome, 00128 Rome, Italy; gianpiero.dantonio@uniroma1.it (G.D.); ginevra.bolino@uniroma1.it (G.B.); letizia.sorace@uniroma1.it (L.S.); lavinia.pellegrini@uniroma1.it (L.P.); nicola.difazio@uniroma1.it (N.D.F.); paola.frati@uniroma1.it (P.F.)

**Keywords:** cancer survivors, health policy, oncological oblivion

## Abstract

Despite the increasing efficacy of modern medicine in diagnosing and treating cancer, survivors often face discrimination in employment, economics, insurance, and society. Law no. 193/2023, also known as the “Oncological Oblivion Law”, aims to provide an initial legislative response to discrimination against cancer survivors in Italy. After defining oncological oblivion in Article 1, the Law provides, in Articles 2, 3, and 4, directives to prevent discrimination against cancer survivors in the area of access to banking and insurance services, adoption procedures and access to or retention in employment. The aim of this work is to illustrate the content and the critical aspects of the recent Law 193/2023 in the landscape of European directives. The legislative process at the Chamber of Deputies and the Senate of the Italian Republic has been retraced through the consultation of preparatory works and bills registered on institutional databases. Law 193/2023 represents the first initiative in Italy aimed at the recognition of the right to oncological oblivion, not only in access to banking and insurance services as in other countries, but also in adoption, employment, and re-employment. Our opinion piece highlights the need for further clarification and expansion to prevent discrimination and protect the social–work–relational rights of people who have been affected by oncological diseases.

## 1. Introduction

Europe accounts for less than 10% of the world’s population, but the current estimate and most recent data suggest that this population represents 25% of the world’s cancer cases. In 2020 alone, the number of cancer-related deaths was 1.9 million, while the number of new diagnoses was about 4 million [1,2,3]. Although less than the rest of the world, the incidence of pediatric and adolescent cancers (i.e., diagnosed in the 0–19 age group) is also significant, with an overall economic impact estimated at approximately EUR 100 billion annually [4]. In Italy alone, the number of new diagnoses in 2023 were about 395,000, a sharp increase from 376,000 in 2022 [5]. This is in part due to the increasingly accurate techniques allowing for early diagnoses as well as the indirect effects of the COVID-19 pandemic. The pandemic saw the inevitable postponing of screening, diagnosis, and early intervention in the oncologic field [6]. Advances in science have, at the same time, reduced mortality; currently, half of all people diagnosed with cancer survive more than 10 years [7]. A recent position paper from the Italian Alliance Against Cancer Survivorship Care Working Group highlighted how for many types of cancer, patients reach a life expectancy similar to that of the general population a few years after treatment [8]. Several studies identify how cancer-related mortality is also declining in Italy, noting that the overall 5-year survival is 51% for males and 62% for females, and the life expectancy for individuals with certain types of neoplasms (breast, prostate), especially if diagnosed at a young age, is equal to that of the general population [9,10].

The projected numbers, paired with the growing public and media concern regarding the increasing incidence of neoplastic diseases in young people, highlight the importance such diseases hold. This importance is not only in terms of public health concern but also the social, relational, and occupational repercussions that such diagnoses have [11,12,13,14]. Indeed, despite being cured, many cancer survivors find themselves discriminated against in different contexts due to the reduced life expectancy it is thought they will have. Examples include accessing a mortgage, taking out an insurance policy, participating in a competition with mental and physical fitness requirements, and adoption procedures. In fact, insurance and mortgage premiums are often increased so much due to the oncological history of patients that they are unaffordable for patients who, because of their disease, have had difficulty with employment. Adoption procedures are made more difficult because of laws requiring a medical history survey for those who request it. Even in the context of public and private competitive practices, the mere fact of having been sick with cancer can be cause for ineligibility and thus result in exclusion. These conditions have in part already been studied internationally; it is known, in fact, that the diagnosis of cancer is associated with the loss of employment or more generally with a decrease in psychological and social functioning [15,16].

The right to oncological oblivion that was promoted (but only partly guaranteed) by the recent Law 193/2023 represents Italy’s response to the social stigma of neoplastic disease in cancer survivors. The aim of this work is to illustrate the content and the critical aspects of Law 193/2023.

The legislative process at the Chamber of Deputies and the Senate of the Italian Republic has been retraced through the consultation of preparatory works and bills registered on institutional databases of the Italian Parliament using the search term “oncological oblivion” and references to Law 193/2023 dating from the first bill promoted (2022) to the promulgation of the Law in the official gazette (2023) [17,18]. An analysis was conducted, from a medico-legal perspective, of the innovations introduced by the Law, also making a comparison with the European landscape in recent years, especially following the European Commission’s 2021 directives. The scope of this search was to discuss among the authors, both in person and online, the contents and criticalities of the Law and its legislative roots, in view of academic discussion and legislative optimization.

## 2. Analysis

### 2.1. Law No. 193/2023

Law No. 193/2023 was published in official gazette No. 294 of 18 December 2023: “Provisions for the prevention of discriminations and protection of the rights of people who have been affected by oncological diseases”, also known as “The right to oncological oblivion” [19].

The Law consists of five articles, structured as summarized in Table 1.

The Law, after introducing the concept of oncological oblivion in Art. 1, gives specific provisions for some of the situations in which people affected in the past by oncological pathology might be disadvantaged. Articles 2, 3, and 4, in addition to delimiting the scope of application of the Law, amend some laws and regulations of the Italian legal system in the area of banking and insurance services (Art. 2), in the area of adoption procedures (Art. 3), and in public and private selections (Art. 4). The same Article 4 promotes policies for reintegration and permanence in employment for people recovered from cancer. The ultimate target of this law is to protect people who have survived oncologic disease, in order to prevent any form of prejudice or different treatment, and to defend every person from public authorities’ interference, unless it is specifically required for the law itself.

Article 2 defines the persons protected by the rule, identifying them as those previously affected and then cured of cancer and whose active treatment (thus excluding follow-up visits) has been concluded without recurrence for at least ten years. So, “recovery” is meant in a temporal sense, in a clinical sense (the absence of recurrence episodes), and in a therapeutic sense (the conclusion of active treatment). With reference to pediatric and juvenile neoplastic diseases, the same article states that the 10-year period is halved if the disease arose before the age of 21. This basic rule applies to the cases provided for in Articles 2, 3, and 4 of the Law, namely, financial, banking, and insurance contracts, adoptions, and selective procedures.

Regarding access to banking, financial, investment, and insurance services, and also in the stipulation of any other type of contract (thus also exclusively between private individuals), the request for information about the policyholder’s oncological medical history is not permitted, nor is it possible to acquire such information when it comes from other sources. Likewise, if this information is in any case available to the operator or financial intermediary, it cannot be used to determine the terms of the contract. In addition, no additional limits, costs, or charges may be applied to subjects protected by the Law, nor may they be treated differently from non-protected subjects. It is also prohibited by Law 193/2023 (Art. 2, paragraph 4) for banks, credit institutions, insurance companies, and financial intermediaries to require the performance of medical checkups and health examinations for the conclusion of contracts from protected persons. If contracts stipulated after the publication of the Law do not comply with the rule, this conflict does not result in the annulment of the whole contract but only of the individual clauses that differ. The right to oncological oblivion is guaranteed by Article 2 even if a specific oncological history was provided before the Law came into force.

Article 3 amends the content of Law No. 184 of the 4th of May 1983, on adoptions [20]. Also, in this case, it is not possible either for the court or for the social welfare services in charge to request information regarding oncological history in the terms already provided in Article 2. This protection is equally granted for national and international requests.

Regarding labor reintegration of individuals cured of neoplastic pathology, the right to oncological oblivion is guaranteed by Article 4 of Law 193 of 2023, dealing with access to public and private competitive and selective procedures, in the event that the requirements contain psychophysical checks or a verification of the candidate’s state of health. Moreover, the second paragraph of the same article provides for the promotion of active policies aimed at all people cured of cancer (who may be outside the above-mentioned time limits). This protection enables inclusion and permanence in the world of work as well as career retraining and salary pathways.

In Article 5 of Law 193 of 2023, the final statements establish that within the first three months of enactment, the Law will define the list of oncological diseases for which shorter time limits, less than those stipulated in the standard through a decree of the Ministry of Health, apply.

### 2.2. European Initiative

The legislative process that gave birth to this Law started from the appeal made by the European Commission to member states through the “European Plan to Combat Cancer” in 2021 [4]. The European initiative was aimed at coordinating the efforts of each member country in the prevention, diagnosis, and treatment of neoplastic diseases. One of the issues addressed by the document is that of improving the quality of life of cancer patients, survivors of the disease, and care providers. Especially, it is emphasized how cancer survivors usually face obstacles in returning to work and in their professional lives after diagnosis and treatment. Furthermore, because of their medical history, cancer survivors receive unequal treatment in terms of access to financial and non-financial services. Obtaining mortgages, taking out insurance policies, and adopting a minor, are far more difficult for people affected by oncological pathologies, due to the high cost of premiums and co-pays or the specific regulations. Hence, there is an appropriate need for professional bodies and associations to provide the “right to oncological oblivion”, referred to precisely by Law 193/2023 [21]. As stated in Paragraph 2 of Article 1, oncological oblivion means “...the right of persons cured of an oncological condition not to disclose information or be subjected to investigation regarding their past medical condition”. As also clarified in Article 1, the provision is also rooted in Article 8 of the European Convention on Human Rights for which “Everyone has the right to protect and respect their private and family life, their home and correspondence” [22]. Also referred to in Article 1 are Articles 2, 3, and 32 of the Italian Constitution as well as Articles 7, 21, 35, and 38 of the European Convention on Human Rights. This is to say that Law 193 is inspired by the fundamental principles of inviolable human rights, nondiscrimination, and health [23].

The Law has its roots in Italy in various bills promoted by the parliament (from right-wing, centrist, and left-wing parties as well as the CNEL—National Council for Economics and Labour) at the Chamber of Deputies starting from the end of 2022 (C413–C690–C744–C885–C959–C1013–C1066–C1182–C1200). These bills later merged into a single bill (C-249) approved and transmitted to the Senate on 3 August 2023 [24]. While the guiding principles of the various bills approved by the Chamber and the Senate, and later of the final law, were the same from the beginning, there were different elements of dialogue between the parties in the various government proposals. Among all, the most debated were the different timeframes for the right to oblivion in the different cases provided by the law and the establishment of an advisory body within the Ministry of Health to oversee the implementation of the law and promote its awareness among banking and insurance operators as well as consumers. The text, as currently in force, was definitively approved on 5 December 2023 [25].

## 3. Discussion

### 3.1. Other European Countries and the Current Law

The right to be forgotten undoubtedly represents the first step toward removing all obstacles that limit a person’s right to protect their private and family life, although there is still a long way to go.

Before Italy, similar legislative measures were also implemented in other European countries. France was the first to establish by law (in January 2016) that people with previously diagnosed cancer disease who have recovered are not required to inform insurers about their previous illness. Consequently, Belgium (April 2019), the Netherlands and Luxembourg (January 2021), and Portugal (January 2022) passed laws to guarantee the right to oncological oblivion. More recently, Spain (July 2023) and Cyprus (November 2023) adopted similar measures, followed by Italy (December 2023) [26,27,28,29,30]. In other major non-European countries, including the UK and the US, although there are systems of protection in the areas of genetic disclosure and disability, there are to date no specific laws or regulations protecting the right to oncological oblivion as provided by Law 193/2023.

The purpose of the legislative measures adopted in all of these countries is the same: to protect those cured of cancer disease from discrimination, especially financial. However, the legislative acts adopted vary depending on the country implementing them, thus making it difficult to propose a common European definition of the right to oncological oblivion. The clinical and therapeutic criteria defined in Law 193/2023 in Italy also apply in the laws promoted on the same subject in other European countries. The major differences concern the temporal criterion. According to the provisions of France, the Netherlands, Luxembourg, Cyprus, and Italy, if the diagnosis of neoplastic pathology was made before the age of 21 years, one has the right to oblivion after five years from the end of treatment, in the absence of disease recurrence. In Romania and Luxembourg, a similar principle applies to individuals whose neoplastic pathology was diagnosed before the age of 18.

Conversely, regarding adults, in Portugal, Belgium, the Netherlands, Cyprus, and Luxembourg, as well as in Italy, it takes 10 years from the end of treatment, in the absence of any relapse of the disease, to have the right to oncological oblivion; in Romania, the same rights are acquired after 7 years, while in France and Spain, 5 years are sufficient [31]. The only exception is Belgium, whose regulations stipulate that persons who have suffered from a neoplastic disease and wish to take out mortgages or loan insurance, must declare this condition. However, after the specified period of no recurrence has elapsed, the insurance company is prohibited from taking this pathology into account when determining the insured person’s current health status, hence ensuring the person’s right to nondiscrimination.

In France, which was the first country to enact a law on oncological oblivion, data from insurance institutions’ reports show that in recent years, the number of contracts in favor of persons protected by the law has increased, amounting to about 10% in 2022 [32]. To the best of our knowledge, there are still no official reports on the socioeconomic effects of similar legal provisions in other European countries.

It is important to consider that, compared to the protections provided by the other states mentioned above, Law 193/2023 includes a broader scope of application. While other legislations focus exclusively on access to banking and insurance services, the Italian law, as mentioned above, also includes the protection of any type of contract, like adoptions, as well as the sphere of employment and re-employment. In strictly legal terms, this coverage results in the forfeiture of penal relevance in the omission of such types of information for all cases indicated by the Law.

### 3.2. Future Directions—LAW 193/2023

The same law identifies the need and expected timeframe for a series of future decrees that will specifically regulate the basic rationale of the rule, which is nonetheless valid and current. Hence, specific forms and modules must be created for the stipulation of contracts, but especially a change in the modalities and forms for the certification of the existence of health requirements so that protected individuals can assert their right to oncological oblivion. Finally, Law 193/2023 stipulates that the Ministry of Health, by special decree, shall define the list of oncological diseases for which terms shorter than the decade or five years already established by the basic norm shall apply. It also establishes that this list be updated annually.

By decree of the Ministry of Health dated 22 March 2024, the first table referred to in Article 5 of Law 193/2023 was published [33]. The list consists of only ten diseases for which the affected anatomical districts, specific details on the age onset of the tumor, the stage of the disease itself, and the years required for the right to be forgotten as defined by the law are considered. In particular, the list highlights stage I colorectal cancers, stage I and II breast cancers, and testicular cancers for which a limit of one year is set. The same limit is set for certain thyroid cancers in women under 55 years old and men under 45 years old. It is hoped that this table will be regularly expanded, in relation to the available and continuously evolving scientific evidence and considering that other neoplastic diseases treated at the initial stages or even in situ have a possibility of recurrence close to zero [34].

Law 193/2023, although not retroactive, certainly represents an important step taken by the Legislature to prevent any form of discrimination that any person might suffer by virtue of their past oncological condition. However, from reading the law, some doubts arise. First, the Law does not refer to the negative outcomes that may arise from the therapeutic procedures that oncological patients undergo in order to heal and recover, as these may represent permanent sequelae that could reduce life expectancy, albeit differently according to the particular case [5,35,36]. Therefore, the question arises as what to do in those scenarios envisaged by the Law, for example, in cases of mutilating surgical outcomes (loss of an organ, resections of the gastrointestinal system, liver resections), or in cases of permanent changes related to the therapy performed (including chemotherapy and radiotherapy) such as lymphedema, hypothyroidism, cardiac or pulmonary problems, asthenia, and nervous and cognitive deterioration. During the medico-legal professional activity of some of the authors, there have been several testimonies of difficulties in accessing financial services for patients who have recovered from oncological pathologies with stabilized outcomes such as permanent ostomy or malabsorption syndromes.

These hypotheses could lead to a situation of inequality between people with the same impairments but resulting from different pathological situations. This observation is also in light of the provisions of Article 25 of Law No. 18 of 2009, the so-called Disability Law, which states “[...] persons with disabilities have the right to enjoy the best possible state of health, without discrimination based on disability [...]” [37]. Nonetheless, the Law does not consider people with a genetic predisposition to the development of neoplastic diseases but who have never received (and may never receive) a cancer diagnosis, as in the cases of BRCA gene mutations, or people undergoing preventive mutilating treatments due to the predisposition they have to developing neoplastic diseases, such as a mastectomy, and therefore might face discrimination in the same areas protected by the Law [38]. In consideration of the principles outlined in Article 3 of the Italian Constitution, which enshrines the principle of equality among all citizens as a fundamental right, the legislator should intervene in this regard. Future clarifications and additions from both the Legislature and the European Commission itself seem desirable.

In addition, considering what has already been achieved in some European countries such as France, Luxembourg, and Belgium, it would be appropriate to hypothesize that the enlargement of those protections, already provided for cancer survivors, may also be extended to people suffering from chronic diseases, such as HIV or HCV, who, thanks to continuous scientific progresses, have their life expectancy increasingly closer to that of healthy subjects. Therefore, it is desirable for the Legislature to make a clear and decisive clarification by taking advantage of the publishment of decrees, so that the right to nondiscrimination does not remain only on paper.

This opinion paper presents several limitations, such as a lack of follow-up on the implementation of legislation and necessary updates of the list of oncological pathologies referred to in Article 5. These suggestions are based on the time limits for the application of oncological oblivion protection, which was a subject of discussion during the preparatory work, accounting for the long period required for its promulgation [24,25].

It is desirable, especially considering the lack of data in the literature on the effect of similar regulations in Europe, to monitor the socioeconomic effects of Law 193 of 2023 over the years. Such monitoring would afford clues for the Legislature regarding future areas of intervention to ensure an effective right to oncological oblivion, as intended by Article 1 of Law 193/2023.

## 4. Conclusions

To summarize, Law 193 of 2023, in the wake of the indications provided by the European Union and work already conducted by other member countries, represents the first initiative in Italy aimed at the recognition of the right to oncological oblivion. Our opinion piece is the first work that illustrates the legislative initiative and highlights, from a medico-legal point of view, some critical issues.

The publication of these decrees in the Law will represent an exceptional opportunity to achieve the target of the Law, giving clear operational indications and integrating as best as possible what has already been established by the norm, namely, the prevention of discrimination and the protection of the social–work–relational rights of people who have been affected by oncological diseases. The considerations regarding the extension of the provisions of Law 193 to other pathologies, as already carried out in other European countries, could be a stimulus for the activity of the Legislature. To all accounts, Law 193/2023, while an important first step, is still not enough to succeed in eliminating the social stigmas of neoplastic disease in cancer survivors.

## Figures and Tables

**Table 1:** Structure of Law 193/2023.

Articles	Title	Key Items
Art. 1	Object, purposes, and definition	Introduces the concept of the right to oncological oblivion and its fundamental principles
Art. 2	Access to banking, financial, investment, and insurance services	Defines the protected individuals under the Law. It establishes the limits of protection regarding access to banking, financial, investment, and insurance services
Art. 3	Amendments to Law No. 184 of the 4th of May 1983, regarding adoption	Applies the protection areas outlined in Art. 2 to both national and international adoption procedures
Art. 4	Access to competitive and selective competitions, employment, and professional training	Applies the protection areas outlined in Art. 2 to both public and private competitive procedures. Promotes active policies to reduce discrimination in the world of employment
Art. 5	Transitional and final provisions	Sets the deadlines for the implementing decrees of the Law, regulates transitional provisions, and ensures the financial non-burden of the law

## Data Availability

No new data were created or analyzed in this study. Data sharing is not applicable to this article.
